# The neutrophil percentage to albumin ratio as a predictor of all-cause and cardiovascular mortality in patients with diabetic kidney disease: A longitudinal cohort analysis of NHANES 2009 to 2018

**DOI:** 10.1097/MD.0000000000047586

**Published:** 2026-02-06

**Authors:** Wang Tao, Yunfeng Yu, Danni Tan, Rong Yu

**Affiliations:** a School of Traditional Chinese Medicine, Hunan University of Chinese Medicine, Changsha, Hunan, China.

**Keywords:** cardiovascular disease mortality, diabetic kidney disease, inflammation, NHANES, The neutrophil percentage to albumin ratio (NPAR) index

## Abstract

The relationship between neutrophil percentage to albumin (NPAR) index and mortality in patients with diabetic kidney disease (DKD) remains unclear. This study aimed to investigate this association by designing a nationally representative longitudinal cohort. We conducted a longitudinal cohort analysis of 1778 adults with DKD from the National Health and Nutrition Examination Survey (2009–2018) with linkage to mortality data through December 31, 2019. Multivariate Cox proportional hazards models were conducted to explore the correlation between the NPAR index and all-cause and cardiovascular disease (CVD) mortality. A restricted cubic spline analysis was performed to explore the potential nonlinear relationship. Additionally, subgroup and sensitivity analysis were conducted to ensure the robustness of the findings. Over a median follow-up of 71 months (84,604 person-years), 462 all-cause and 146 CVD deaths occurred. Compared to the lowest quartile, participants in the highest NPAR quartile had significantly increased risks of all-cause (hazard ratio 1.96, 95% confidence interval 1.49–2.58, *P* < .001) and CVD mortality (hazard ratio 3.62, 95% confidence interval 2.04–6.41, *P* < .001). Restricted cubic spline analysis revealed a J-shaped relationship between NPAR and all-cause mortality (*P* for nonlinearity < .001), whereas NPAR showed a linear positive association with CVD mortality (*P* for nonlinearity = .4771). These findings remained consistent across subgroup and sensitivity analysis. Higher NPAR is independently associated with increased risks of all-cause and cardiovascular mortality in adults with DKD, supporting its potential role as a prognostic biomarker.

## 1. Introduction

With the growing prevalence of diabetes attributed to demographic shifts, aging populations, obesity, and sedentary lifestyles, diabetes has become the third most prevalent chronic disease globally, following cardiovascular and cerebrovascular diseases and malignancies.^[1]^ Diabetic kidney disease (DKD), as a prevalent microvascular complication of diabetes, accounts for about 30% to 40% of diabetic patients and serves as the fundamental cause of chronic kidney disease and end-stage renal disease both in the United States and worldwide.^[2]^ Notably, most patients with DKD are susceptible to infections and cardiovascular disease (CVD) during renal replacement therapy rather than end-stage renal disease alone.^[3]^ According to reports,^[4]^ diabetic patients with renal impairment or elevated urine albumin (ALB) excretion had a 10-year cardiovascular mortality risk of >10%.

The primary process driving the occurrence and progression of DKD is chronic inflammation, which serves as both a biomarker and a therapeutic target for reducing mortality associated with DKD.^[5]^ Neutrophil count and serum ALB levels have been shown in numerous studies^[6–8]^ to be independent predictors of DKD incidence and cardiovascular mortality. Notably, a cross-sectional study indicated that^[9]^ the neutrophil count is the most pivotal independent risk contributor for the progression of DKD. The neutrophil percentage-to-albumin ratio (NPAR) index, represents the ratio of neutrophil percentage to ALB, has drawn interest lately as a potential new indicator of inflammatory and immunologic conditions.^[10]^ Strong correlations have been established by Liu et al^[11]^ between the NPAR index and a higher risk of DKD. Other studies have indicated that NPAR can serve as a predictor for mortality in patients with diabetes or prediabetes.^[12]^

Despite these findings, it remains uncertain whether the NPAR index could predict mortality specifically among patients with DKD, and prospective evidence is still limited. Considering the substantial inflammatory burden and the markedly elevated cardiovascular risk inherent to this population, identifying simple and reliable prognostic markers is of considerable clinical importance. To address this gap, we conducted a nationally representative longitudinal cohort study using data from the National Health and Nutrition Examination Survey (NHANES) to examine the association between NPAR and long-term mortality in adults with DKD. Based on the established links between inflammation, neutrophil activity, and nutritional status, we anticipated that higher NPAR levels would be associated with increased risks of both all-cause and cardiovascular mortality.

## 2. Materials and methods

### 2.1. Study population and design

**Figure 1.**
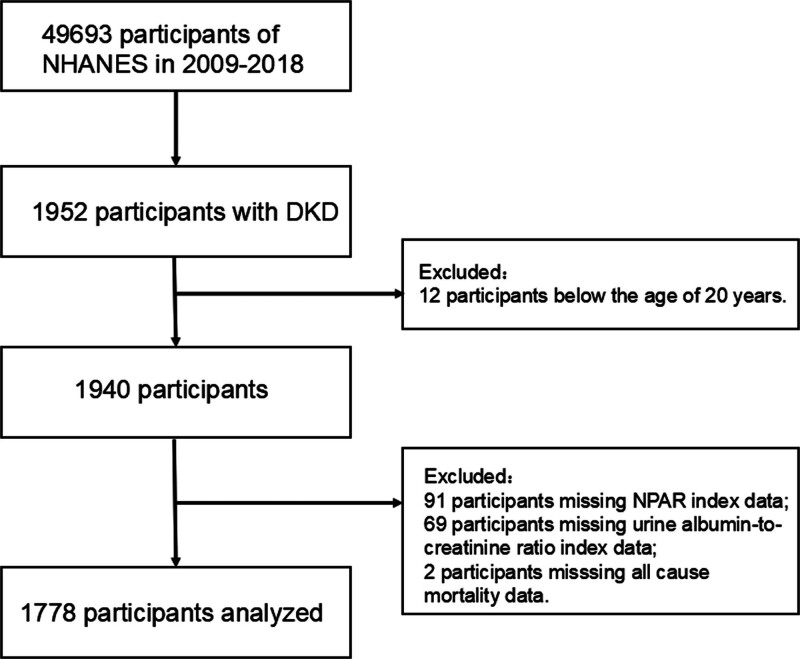
Flow chart of study participants.

The NHANES database, conducted by the National Center for Health Statistics within the Centers for Disease Control and Prevention, is an all-round initiative assessing the well-being elements and nutrition profiles of the USA. It employs a unique approach integrating interviews, physical assessments, and laboratory analyses to gather diverse health data. We obtained signed informed consent forms from all participants involved in the study. Then, data with a total of 1778 participants for the 5 survey cycles spanning from 2009 to 2018 were derived from the NHANES website (https://wwwn.cdc.gov/nchs/nhanes). Eligible DKD patients aged ≥20 years were enrolled, excluding pregnant individuals or those lacking lab data or mortality records (Fig. 1).

### 2.2. Definition of diabetic kidney disease (DKD)

Diabetes was defined based on any of these four criteria: admitted diagnosis of diabetes; use of insulin or oral hypoglycemic medications; fasting plasma glucose (FPG) levels ≥7.0 mmol/L; glycohemoglobin (HbA1c) levels ≥6.5%. The estimated glomerular filtration rate (eGFR) was calculated by the Chronic Kidney Disease Epidemiology Collaboration formula, which is widely recognized for its accuracy in assessing kidney function.^[13]^ The formula is defined as follows: eGFR = 141×($\frac{min\left(\frac{scr}{\kappa },1\right)}{\alpha }$) ×$\frac{max\left(\frac{scr}{\kappa },1\right)}{-1.209}$×0.993^age^ × 1.018 × race coefficient (where *κ* is 0.7 for women and 0.9 for men, *α* is ‐0.329 for women and ‐0.411 for men, and the race coefficient is 1.159 for Black individuals and 1.000 for those who are non-Black). Diabetic individuals with an eGFR <60 mL/min/1.73 m^2^ or a urine albumin-to-creatinine ratio ≥30 mg/g were diagnosed with DKD.

### 2.3. Calculation of the NPAR index

The NPAR index was computed as neutrophil percentage (%)/ALB (g/dL). All data were obtained from the NHANES database. Participants were categorized into 4 quartiles (Q1–Q4) according to their NPAR index, with the lowest quartile (Q1) acting as the reference group.

### 2.4. Ascertainment of mortality

NHANES links to the National Death Index for mortality data, enabling detailed analysis of health outcomes related to mortality trends. Mortality status was obtained from the NHANES public through a linked mortality file, updated as of December 31, 2019. All-cause mortality refers to death from all possible causes, encompassing heart disease (codes 054-068), cerebrovascular diseases (code 070), malignancies (codes 019-043), diabetes (code 046), and other reasons. Deaths due to heart disease were classified as CVD mortality.

### 2.5. Assessment of covariates

From the NHANES website, we gathered a range of demographic, laboratory, examination, and questionnaire data. The body mass index (BMI) was determined by dividing weight in kilograms by the square of height in meters and was assigned into 2 categories: normal (<25 kg/m^2^) and overweight (≥25 kg/m^2^). Race was classified as non-Hispanic Black, non-Hispanic White, Mexican American, or other. The education level was grouped into some college or above, or high school graduate or equivalent, less than high school. Smoking status was recorded as current, never, or former, depending on whether individuals had smoked 100 or more cigarettes in their lifetime and if they were currently smoking. If there was no alcohol consumption or zero frequency during the previous 12 months, the intake was recorded as non-drinking; otherwise, it was recorded as drinking. Hypertension status was determined by a history of high blood pressure, oral antihypertensive medications, or a non-same-day average of at least 4 systolic blood pressure recordings ≥140 mm Hg or diastolic blood pressure recordings ≥90 mm Hg. Hyperlipidemia was described as prescribed cholesterol-loweringdrugs. History of diabetes was characterized by a response of “yes” to the question “Close relative had diabetes?” CVD was diagnosed if the individual reported having chronic heart failure, artery disease, chest pain, myocardial infarction, or cerebrovascular accident (stroke). Clinical indicators including FPG, HbA1c, serum creatinine, uric acid, alanine aminotransferase, aspartate aminotransferase, gamma-glutamyl transaminase, ALB, total cholesterol (TC), triglycerides (TG), low-density lipoprotein cholesterol (LDL-C), and high-density lipoprotein cholesterol were recorded in the NHANES laboratory.

### 2.6. Assessment of missing data

For missing covariate data, we employed multiple imputation by chained equations using the R package “mice” to handle missing data. A total of 5 imputed datasets were generated, with predictive mean matching applied as the imputation method for all continuous variables. The multiple imputation by chained equations algorithm was run for 5 iterations, and convergence was confirmed by visually inspecting the trace plots. The proportion of missing values was as follows: BMI (2.8%), fasting glucose (48.7%), glycohemoglobin (0.1%), triglycerides (49.5%), and LDL-C (52.0%). The quality of the imputations was assessed by comparing the distributions of the observed and imputed values (Fig. S1, Supplemental Digital Content, https://links.lww.com/MD/R353).

### 2.7. Statistical analysis

Statistical analysis was carried out using R software version 4.4.1 (https://www.r-project.org). Considering the complex sampling design of NHANES, we incorporated sample weights, clustering, and stratification in our analysis. Continuous variables were represented as mean and standard deviation, and baseline characteristics across NPAR quartiles were compared by One-way ANOVA. While, categorical variables were presented as frequency and percentage (%), and they were assessed using Pearson Chi-square test.

Multivariable Cox proportional hazards regression models were used to assess the association between NPAR and mortality. The confounders were chosen based on prior literature and included demographic factors (age, sex, BMI, education level, and race), lifestyle factors (smoking and alcohol use), key clinical comorbidities (hypertension, hyperlipidemia, and cardiovascular disease), and major laboratory parameters (FPG, HbA1c, TG, TC, LDL-C, high-densitylipoprotein cholesterol, and uric acid). These factors are well-established risk factors for diabetic kidney disease and mortality.^[14]^ Especially, we evaluated multicollinearity among covariates using variance inflation factors, with a variance inflation factors >10 considered indicative of severe collinearity; highly correlated variables were excluded from final models.

Subsequently, we employed restricted cubic splines and smooth curve fitting (using the penalized spline method) to further investigate the relationships between the NPAR index and mortalities. If nonlinearity was detected, we assessed the potential nonlinear relationship between NPAR and all-cause mortality using bootstrap validation and formal model comparison with Akaike Information Criterion (AIC). Subgroup and sensitivity analysis were also performed to assess consistency of associations. A two-sided *P*-value <.05 was considered statistically significant.

## 3. Results

### 3.1. Baseline characteristics

The baseline characteristics of the 1778 participants, stratified by the NPAR index, are presented in Table 1. The mean age of the participants was 65.38 years, with 54.0% being male. The average NPAR index for enrolled patients was 1.51 ± 0.3. Individuals with a higher NPAR index tended to be older, obese, and have comorbidities such as hypertension, hyperlipidemia, or a history of diabetes. Moreover, significant differences in biochemical parameters were observed among the groups. Individuals in the highest quartile (Q4) exhibited significantly lower eGFR, TC, and TG, alongside increased serum creatinine and uric acid, compared to those in the first quartile (Q1).

**Table 1:** Baseline characteristics according to the NPAR index quartiles.

Characteristics	Quartiles of NPAR index					*P* value
	Overall	Q1 (Q1 ≤ 1.32)	Q2 (1.32 < Q2 ≤ 1.51)	Q3 (1.51 < Q3 ≤ 1.69)	Q4 (Q4>1.69)	
N (%)	1778	445	446	442	445	
Age, years, mean (SD)	65.38 (12.65)	64.71 (12.56)	64.74 (12.50)	65.37 (12.47)	66.71 (13.01)	.062
Gender, n (%)						
Female	817 (46.0)	203 (45.6)	197 (44.2)	199 (45.0)	218 (49.0)	.494
Male	961 (54.0)	242 (54.4)	249 (55.8)	243 (55.0)	227 (51.0)	
BMI, kg/m^2^, mean (SD)	32.52 (7.45)	30.72 (6.28)	32.24 (7.09)	33.40 (7.82)	33.71 (8.11)	**<.001**
BMI, kg/m^2^, n (%)						
<25	241 (13.6)	68 (15.3)	61 (13.7)	52 (11.8)	60 (13.5)	.503
≥25	1537 (86.4)	377 (84.7)	385 (86.3)	390 (88.2)	385 (86.5)	
Race, n (%)						
Mexican American	296 (16.6)	63 (14.2)	76 (17.0)	73 (16.5)	84 (18.9)	**<.001**
Non-Hispanic White	622 (35.0)	96 (21.6)	152 (34.1)	182 (41.2)	192 (43.1)	
Non-Hispanic Black	479 (26.9)	180 (40.4)	110 (24.7)	99 (22.4)	90 (20.2)	
Other	381 (21.4)	106 (23.8)	108 (24.2)	88 (19.9)	79 (17.8)	
Education, n (%)						
Less than high school	627 (35.3)	167 (37.5)	160 (35.9)	151 (34.2)	149 (33.5)	.733
High school grad or equivalent	414 (23.3)	98 (22.0)	102 (22.9)	99 (22.4)	115 (25.8)	
Some college or above	737 (41.5)	180 (40.4)	184 (41.3)	192 (43.4)	181 (40.7)	
Smoking status						
Now	262 (14.7)	65 (14.6)	68 (15.2)	61 (13.8)	68 (15.3)	.221
Never	851 (47.9)	236 (53.0)	209 (46.9)	206 (46.6)	200 (44.9)	
Former	665 (37.4)	144 (32.4)	169 (37.9)	175 (39.6)	177 (39.8)	
Alcohol, n (%)						
Yes	602 (33.9)	157 (35.3)	156 (35.0)	164 (37.1)	125 (28.1)	**.025**
No	1176 (66.1)	288 (64.7)	290 (65.0)	278 (62.9)	320 (71.9)	
Hypertension, n (%)						
Yes	1464 (82.3)	369 (82.9)	364 (81.6)	364 (82.4)	367 (82.5)	.966
No	314 (17.7)	76 (17.1)	82 (18.4)	78 (17.6)	78 (17.5)	
Hyperlipemia						
Yes	1105 (62.1)	279 (62.7)	268 (60.1)	283 (64.0)	275 (61.8)	.671
No	673 (37.9)	166 (37.3)	178 (39.9)	159 (36.0)	170 (38.2)	
History of diabetes						
Yes	1127 (63.4)	285 (64.0)	280 (62.8)	287 (64.9)	275 (61.8)	.779
No	651 (36.6)	160 (36.0)	166 (37.2)	155 (35.1)	170 (38.2)	
Cardiovascular disease						
Yes	561 (31.6)	107 (24.0)	128 (28.7)	156 (35.3)	170 (38.2)	**<.001**
No	1217 (68.4)	338 (76.0)	318 (71.3)	286 (64.7)	275 (61.8)	
Creatinine µmol/L, mean (SD)	109.59 (73.78)	97.47 (43.60)	101.77 (65.10)	112.71 (75.61)	126.44 (97.23)	**<.001**
eGFR,mL/min/1.73 m^2^, mean (SD)	69.43 (30.20)	73.62 (29.45)	74.02 (29.98)	67.95 (29.92)	62.11 (29.95)	**<.001**
eGFR, n (%)						
30 < eGFR ≤ 60 mL/min/1.73 m^2^	116 (6.5)	15 (3.4)	21 (4.7)	32 (7.2)	48 (10.8)	**<.001**
eGFR ≤ 30 mL/min/1.73 m^2^	1662 (93.5)	430 (96.6)	425 (95.3)	410 (92.8)	397 (89.2)	
UACR, mg/g, n (%)						
30 < UACR ≤ 300	1428 (80.3)	387 (87.0)	365 (81.8)	350 (79.2)	326 (73.3)	**<.001**
UACR > 300	350 (19.7)	58 (13.0)	81 (18.2)	92 (20.8)	119 (26.7)	
Uric acid, µmol/L, mean (SD)	369.08 (104.04)	360.76 (92.04)	359.72 (96.17)	371.70 (102.42)	384.19 (121.53)	**.001**
ALT, U/L, mean (SD)	25.37 (36.28)	26.31 (17.44)	26.33 (19.43)	23.69 (16.55)	25.16 (65.65)	.666
AST, U/L, mean (SD)	25.98 (20.37)	26.97 (14.46)	26.22 (15.85)	25.57 (16.03)	25.16 (30.71)	.571
GGT, U/L, mean (SD)	38.73 (47.96)	37.05 (43.35)	37.93 (45.03)	40.43 (50.96)	39.50 (52.02)	.72
Glucose,mean (SD)	9.40 (3.91)	9.04 (3.73)	9.45 (3.97)	9.39 (3.78)	9.71 (4.12)	.085
Glycohemoglobin, %, mean (SD)	7.64 (1.93)	7.49 (1.85)	7.62 (1.94)	7.61 (1.80)	7.84 (2.10)	**.048**
TC, mmol/L,mean (SD)	4.68 (1.25)	4.87 (1.30)	4.70 (1.25)	4.70 (1.26)	4.47 (1.14)	**<.001**
TG, mmol/L, mean (SD)	2.31 (2.39)	2.51 (2.57)	2.31 (2.44)	2.52 (2.71)	1.91 (1.66)	**<.001**
LDLC, mmol/L, mean (SD)	2.42 (0.98)	2.49 (0.99)	2.46 (1.03)	2.36 (0.98)	2.36 (0.93)	.096
HDL-C, mmol/L, mean (SD)	1.23 (0.39)	1.26 (0.40)	1.21 (0.32)	1.23 (0.44)	1.24 (0.39)	.36
Albumin, g/L, mean (SD)	40.42 (3.71)	42.42 (3.15)	41.50 (2.97)	40.38 (2.93)	37.38 (3.67)	**<.001**
NEUT, %, mean (SD)	60.54 (9.83)	48.70 (7.27)	58.87 (4.51)	64.31 (4.79)	70.31 (6.13)	**<.001**
NPAR, mean (SD)	1.51 (0.30)	1.15 (0.15)	1.42 (0.05)	1.59 (0.05)	1.89 (0.22)	**<.001**

Continuous variables were expressed as mean ± SD, and categorical variables were presented as percentages with 95% confidence intervals. Bold value indicates statistical significance.

ALT = alanine aminotransferase, AST = aspartate aminotransferase, eGFR = estimated glomerular filtration rate, GGT = gamma glutamyl transaminase, HDL-C = high-density lipoprotein cholesterol, NEUT% = neutrophil percentage, NPAR = neutrophil percentage to albumin, SD = standard deviation, TG = triglycerides, UACR = urine albumin to creatinine ratio.

### 3.2. Relationships between the NPAR index and mortality

Table 2 shows the incidence of 462 from all causes and 146 deaths related to CVD over a median follow-up of 71 months. We constructed three Cox regression models to evaluate the relationships between the NPAR index and mortality risk. Above all, we did not adjust for any confounding factors in Model 1, and the NPAR index showed a positive correlation with a higher risk of CVD mortality among patients with DKD. After adjusting for various covariates in Model 3, participants in the highest quartile (Q4) of the NPAR index had an elevated risk of all-cause mortality (hazard ratio [HR] 1.96, 95% confidence interval [CI] 1.49–2.58, *P* < .001) and CVD mortality (HR 3.62, 95% CI 2.04–6.41, *P* < .001) compared to those in the first quartile (all *P* for trend < .05).

**Table 2:** HRs (95% CIs) for mortality according to the index quartiles.

	Q1 (Q1 ≤ 1.32)	Q2 (1.32 < Q2 ≤ 1.51)	Q3 (1.51 < Q3 ≤ 1.69)	Q4 (Q4 > 1.69)	*P* trend
**All-cause mortality (N = 462**)					
Model 1 HR (95% CI) *P*-value	1	1.14 (0.85–1.53), *P* = .375	1.56 (1.18–2.07), ***P* = .002**	2.45 (1.88–3.18), ***P* < .001**	***P* < .001**
Model 2 HR (95% CI) *P*-value	1	1.11 (0.82–1.49), *P* = .498	1.49 (1.12–1.98), ***P* = .006**	2.29 (1.75–3.00), ***P* < .001**	***P* < .001**
Model 3 HR (95% CI) *P*-value	1	1.08 (0.80–1.46), *P* = .599	1.27 (0.95–1.69), ***P* = .103**	1.96 (1.49–2.58), ***P* < .001**	***P* < .001**
**CVD-mortality (N = 146**)					
Model 1 HR (95% CI) *P*-value	1	2.38 (1.32–4.28), ***P* = .004**	2.38 (1.31–4.31), ***P* = .004**	4.58 (2.64–7.97), ***P* < .001**	***P* < .001**
Model 2 HR (95% CI) *P*-value	1	2.30 (1.27–4.16), ***P* = .006**	2.24 (1.23–4.09), ***P* = .009**	2.24 (1.23–4.09), ***P* = .009**	***P* < .001**
Model 3 HR (95% CI) *P*-value	1	2.32 (1.28–4.19), ***P* = .006**	1.89 (1.03–3.47), ***P* = .039**	3.62 (2.04–6.41), ***P* < .001**	***P* < .001**

Model 1 was non-adjusted. Model 2 was adjusted for age, gender, BMI, race, education, tobacco use, and alcohol use. Model 3 was adjusted for age, gender, BMI, race, education, tobacco use, alcohol use, hypertension, hyperlipidemic, cardiovascular disease, history of diabetes, glucose, glycohemoglobin, UA, TC, TC, HDLC, LDLC. Bold value indicates statistical significance.

BMI = body mass index, CVD = cardiovascular disease, HDL-C = high-density lipoprotein cholesterol, HR = hazard ratio, LDL-C = low-density lipoprotein cholesterol, TC = total cholesterol, UA = uric acid.

### 3.3. Exploration of nonlinear relationships

**Figure 2.**
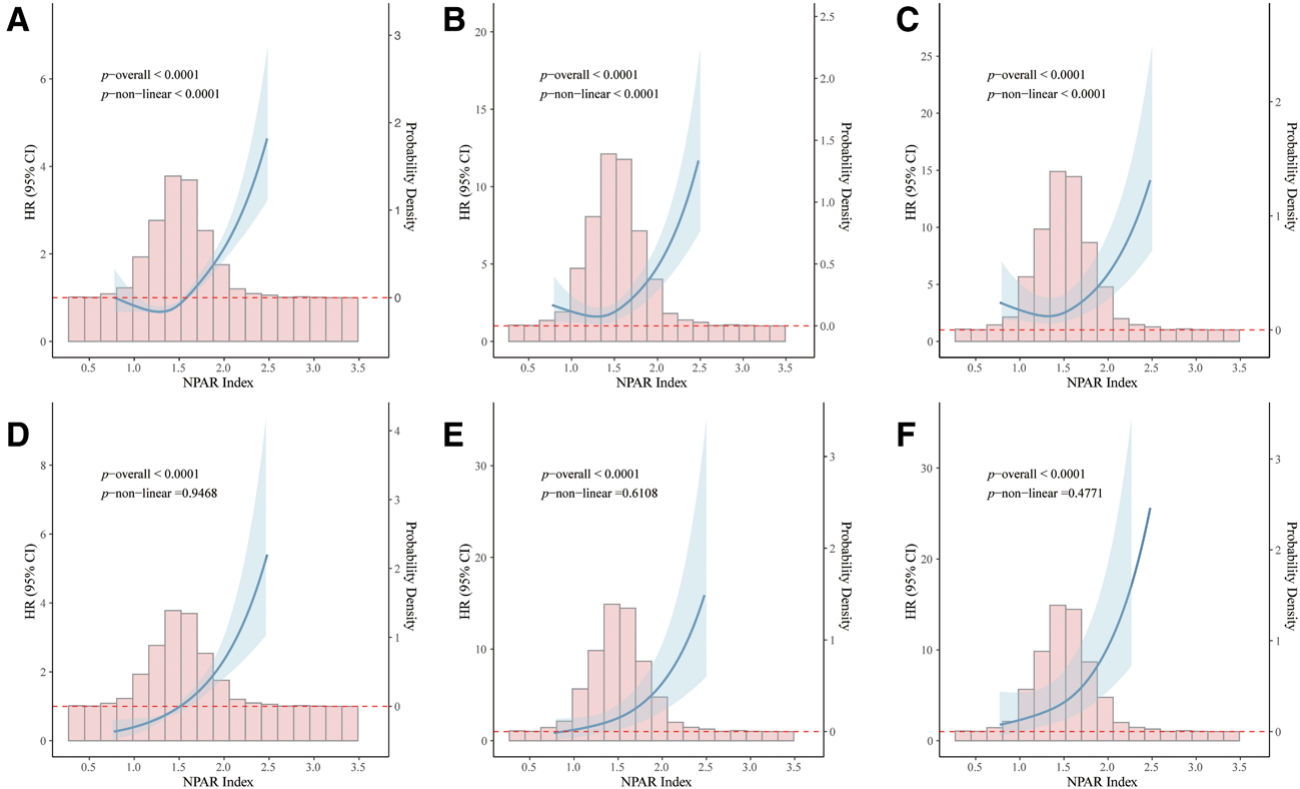
The RCS analysis between the NPAR index and the all-cause mortality (A–C) and CVD mortality (D–F). Subplots A to C present results for all-cause mortality, while subplots D to F correspond to CVD mortality. Each row depicts model adjustments as follows: unadjusted (A, D), partially adjusted (B, E), and fully adjusted (C, F). The blue solid curve denotes the hazard ratio (HR) estimated across the range of NPAR values. Surrounding this line, the light-blue band indicates the 95% confidence interval (CI). The red dashed line marks the reference line (HR = 1). The distribution of NPAR within the study sample is shown via histogram at the bottom of each plot. *P*-values indicating overall association and for nonlinearity are provided in the top-left section of each panel. The partially adjusted model included age, gender, BMI, race, education, tobacco use and alcohol use. The fully adjusted model further included hypertension, hyperlipidemia, cardiovascular disease, history of diabetes, glucose, glycohemoglobin, UA, TC, TC, HDLC, LDLC. BMI = body mass index, CVD = cardiovascular disease, HDL-C = high-density lipoprotein cholesterol, HR = hazard ratio, LDLC = low-density lipoprotein cholesterol, NPAR = neutrophil percentage to albumin, RCS = restricted cubic spline, TC = total cholesterol, UA = uric acid.

Multivariable Cox regression analysis revealed a positive correlation between the NPAR index and mortalities among patients with DKD. To further investigate this relationship, we employed a Cox proportional hazards regression model with restricted cubic spline (RCS) and smooth curve fitting (penalty spline method). Remarkably, after controlling for nearly all variables, a striking J-shaped relationship was evident between the NPAR index and all-cause mortality (*P *< .001 for nonlinearity, Fig. 2A–C). In contrast, the NPAR index exhibited a linear positive correlation with cardiovascular mortality (*P* = .477 for nonlinearity, Fig. 2D–F).

Although RCS analysis suggested a potential nonlinear trend between NPAR and all-cause mortality, and the maximum likelihood threshold identified by a grid search method was at 1.09, further bootstrap validation indicated that this threshold estimate was unstable (95% CI: 1.00–1.58). Simultaneously, model comparison based on the AIC demonstrated that the goodness-of-fit of the linear model (AIC = 73,406,735) was significantly superior to that of the threshold-based piecewise model (AIC = 84,509,335; ΔAIC > 11,000,000). In summary, the current evidence more strongly supports the existence of a continuous, linear dose–response relationship between NPAR and the risk of all-cause mortality.

### 3.4. Subgroup and sensitivity analysis

**Figure 3.**
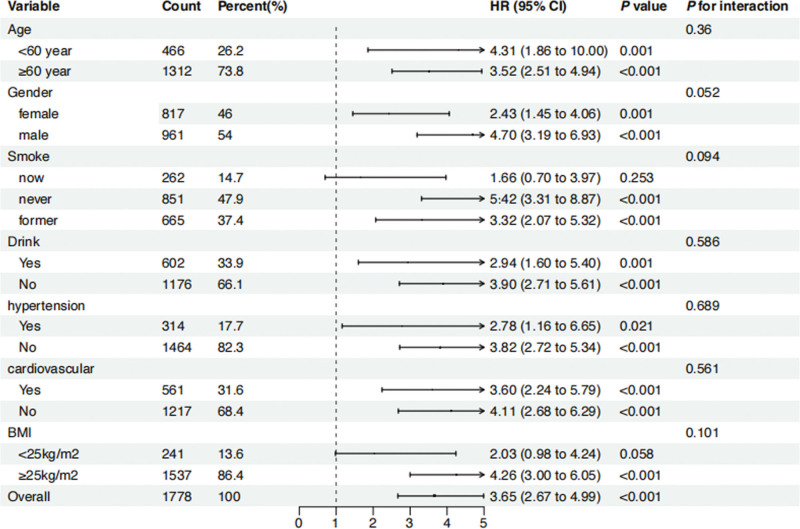
Forest plot of stratified analyses of NPAR and all-cause mortality in patients with DKD. HRs adjusted for age, gender, BMI, race, education, tobacco use, alcohol use, hypertension, hyperlipidemic, cardiovascular disease, history of diabetes. BMI = body mass index.

**Figure 4.**
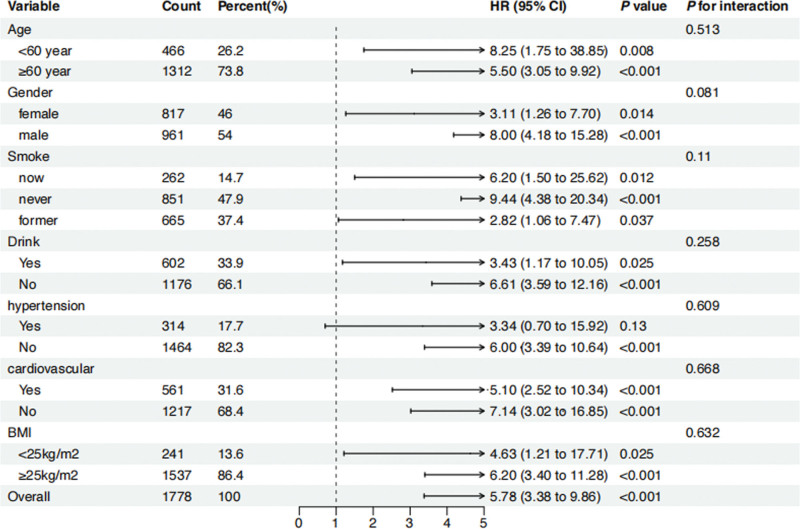
Forest plot of Stratified analyses of NPAR and CVD mortality in patients with DKD. HRs adjusted for age, gender, BMI, race, education, tobacco use, alcohol use, hypertension, hyperlipidemic, cardiovascular disease, history of diabetes. BMI = body mass index, CVD = cardiovascular disease, DKD = diabetic kidney disease, HR = hazard ratio.

Subgroup and interaction analyses were conducted to ensure the stability of the results. We examined whether the NPAR index predicts mortalities differently across various subgroups, including age, sex, BMI, smoking, alcohol, hypertension, and cardiovascular disease. The results showed that, except for smokers, those with a BMI < 25 kg/m^2^, and those with hypertension, the NPAR index was positively correlated with all-cause mortality (Fig. 3) and CVD mortality in other subgroups (Fig. 4). And it is noteworthy that there was no evidence of interaction between baseline NPAR index and stratification variables (*P* > .05).

To further assess the robustness of our primary findings, we performed multiple sensitivity analyses. A complete-case analysis (Table S1, Supplemental Digital Content, https://links.lww.com/MD/R353) and an analysis excluding participants with extreme NPAR values (Table S2, Supplemental Digital Content, https://links.lww.com/MD/R353) both yielded results consistent with the main analysis, confirming that the association between NPAR and mortality remained stable under different data inclusion criteria and modeling assumptions (*P* < .05).

## 4. Discussion

Our research demonstrates that in individuals with DKD, the NPAR index is a reliable and robust indicator of death from CVD and all causes. Although RCS analysis initially suggested a potential nonlinear association with all-cause mortality, this finding was not stable upon rigorous statistical validation using bootstrap resampling and the AIC comparisons. Therefore, the current evidence supports a linear relationship between NPAR and both all-cause and cardiovascular mortality in patients with DKD. This conclusion is further reinforced by the consistency of results across extensive sensitivity and subgroup analysis.

CVD remains the leading cause of death in patients with DKD. Our findings align with previous studies,^[15]^ which report that approximately 31.6% of DKD patients succumb to CVD. Hu et al^[16]^ found that higher NPAR levels were linked to a greater incidence of hospitalization, as well as 90-day and 365-day mortality in heart failure patients. Our work adds to the body of evidence demonstrating the NPAR’s superior predictive capability for both all-cause and CVD mortality in patients with DKD. The risk of CVD mortality was 3.62 times higher for patients in the highest quartile of the NPAR score (HR 3.62, 95% CI 2.04–6.41, *P* < .001). The potential mechanisms underlying this relationship may involve inflammatory responses.^[17]^ Inflammation has been identified in numerous studies^[18,19]^ as a shared risk factor for both DKD and CVD, promoting atherosclerosis, increasing plaque susceptibility, inducing endothelial dysfunction, and damaging renal tubules. Although the exact causes of tubulointerstitial injury in DKD remain unclear, inflammation is acknowledged as a critical factor in tubular damage.^[20]^

Neutrophils, recognized as the initial line of defense in the innate immune system,^[21]^ migrate swiftly to infection sites through mechanisms such as phagocytosis, degranulation, and the release of reactive oxygen species and neutrophil extracellular traps to eliminate invading pathogens.^[22]^ Renal biopsies from patients with DKD often reveal notable infiltration by neutrophils, macrophages, and lymphocytes.^[23]^ Serum ALB, a marker of nutritional and immune status, serves as a potent independent prognostic factor for CVD events.^[24]^ The NPAR index thus offers a composite measure of systemic inflammatory and nutritional status by integrating 2 routine clinical indicators. This is supported by its positive correlation with hs-CRP levels.^[25]^ Additionally, our finding that the NPAR index is positively associated with serum uric acid and creatinine levels suggests it reflects not only inflammation but also renal function and overall disease severity in DKD. Together, these associations imply that NPAR provides valuable insight into the integrated health status and prognosis of patients with DKD.

Moreover, the NPAR index holds immediate clinical value due to its simplicity and low cost, as it is derived from routine blood tests. It provides a practical tool for risk stratification in patients with DKD. An elevated NPAR could help identify high-risk individuals requiring closer monitoring or earlier intervention, while serial measurements may track disease progression or treatment response. Future prospective validation could enable its integration into prognostic models, promoting more personalized management.

The primary strength of our research lies in its substantial sample size and extended follow-up period, with all participants drawn from the NHANES database, ensuring that selection bias is minimized through weighted calculations. However, several limitations must be noted. Firstly, the study predominantly focuses on the American population, casting doubt on the findings’ applicability to different populations. Secondly, although we accounted for potential confounders in our analysis, the influence of other unmeasured factors on the NPAR index cannot be entirely excluded. Lastly, our assessment was limited to the predictive value of baseline NPAR. Both neutrophil percentage and serum ALB are subject to biological variability and can be influenced by transient conditions such as acute inflammation or nutritional status. This reliance on a single time-point measurement may introduce measurement bias. We explicitly recommend that future longitudinal studies with repeated measurements are warranted to validate our findings and to more accurately assess the long-term prognostic value of NPAR.

## 5. Conclusion

Our study demonstrates that a high NPAR index is a standalone indicator of both all-cause and CVD mortality in patients with DKD. Notwithstanding these strengths, it is essential to acknowledge the study’s limitations, including residual confounding factors and the potential inaccuracies associated with a single baseline NPAR measurement. Therefore, further external validation in large-scale, longitudinal studies with repeated measurements is required before NPAR could be considered for clinical application.

## Acknowledgments

The authors gratefully acknowledge the National Health and Nutrition Examination Survey (NHANES) for providing the data on the health and nutritional status of the national population.

## Author contributions

**Conceptualization:** Wang Tao, Yunfeng Yu.

**Data curation:** Wang Tao, Yunfeng Yu, Danni Tan.

**Funding acquisition:** Rong Yu.

**Investigation:** Wang Tao, Danni Tan.

**Methodology:** Wang Tao, Yunfeng Yu.

**Project administration:** Rong Yu.

**Supervision:** Rong Yu.

**Visualization:** Wang Tao.

**Writing – original draft:** Wang Tao, Danni Tan.

**Writing – review & editing:** Yunfeng Yu, Rong Yu.

## Supplementary Material


