# Supplementary material for: The neutrophil percentage to albumin ratio as a predictor of all-cause and cardiovascular mortality in patients with diabetic kidney disease: A longitudinal cohort analysis of NHANES 2009 to 2018
Source: Medicine (Baltimore). 2026 Feb 6;105(6):e47586. doi: 10.1097/MD.0000000000047586 (PMC12885741; doi:10.1097/MD.0000000000047586)
Supplement: Supplementary file 1 [file medi-105-e47586-s001.docx]

**Supplementary Fig1.** Density plots comparing the distributions of observed and imputed values for key continuous covariates. The blue curves represent the imputed data, and the red curves represent the original distribution.


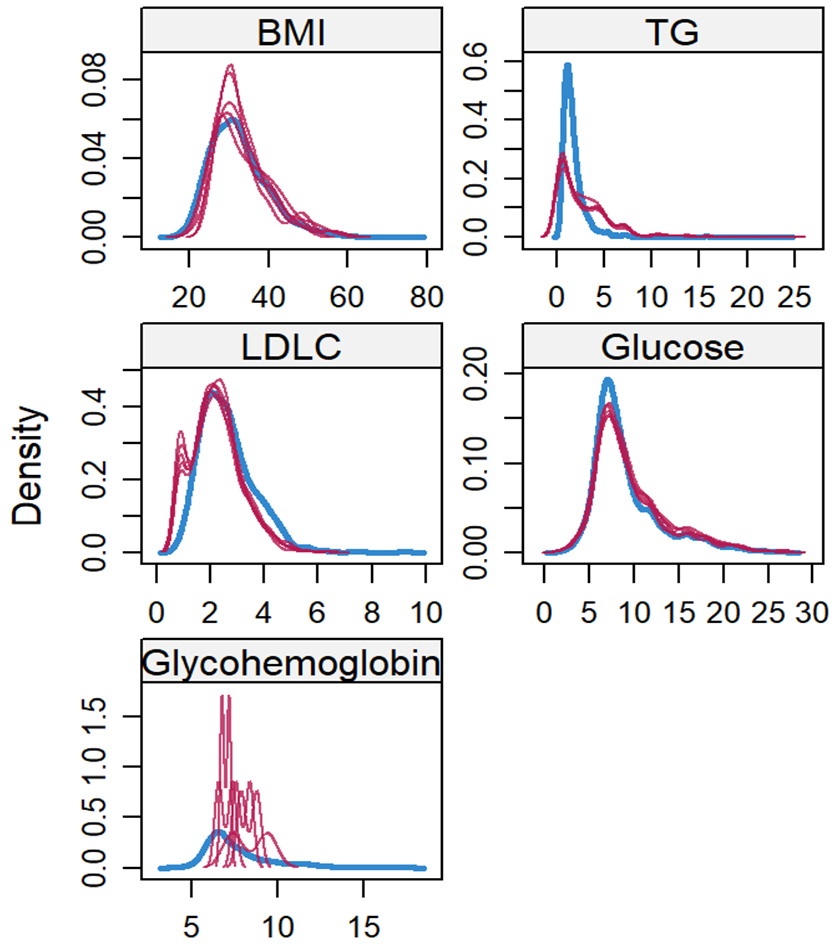


**Supplementary Table 1 Complete-case sensitivity analysis of NPAR**

|  | Model 1  HR (95% CI) *P-*value | Model 2  HR (95% CI) *P-*value | Model 3  HR (95% CI) *P-*value |
| --- | --- | --- | --- |
| **All-cause mortality (N=462)** | |  |  |
| NPAR_complete-case | 3.76 (2.49-5.66),  ***p<*0.0001** | 3.59 (2.28-5.65),  ***p<*0.0001** | 2.89 (1.78-4.70),  ***p<*0.0001** |
| **CVD-mortality (N=146)** | |  |  |
| NPAR_complete-case | 4.87 (2.71-8.77),  ***p<*0.0001** | 4.88 (2.39-9.96),  ***p<*0.0001** | 4.26 (1.98-9.17),  ***p*=0.0002** |

Model 1 was non-adjusted. Model 2 was adjusted for age, gender, BMI, race, education, tobacco use, and alcohol use. Model 3 was adjusted for age, gender, BMI, race, education, tobacco use, alcohol use, hypertension, hyperlipidemic, cardiovascular disease, history of diabetes, FPG, HbA1c, UA, TC, TC, HDLC, LDLC. Bold value indicates statistical significance.

**Supplementary Table 2 Sensitivity analysis after excluding extreme values of NPAR**

|  | Model 1  HR (95% CI) *P-*value | Model 2  HR (95% CI) *P-*value | Model 3  HR (95% CI) *P-*value |
| --- | --- | --- | --- |
| **All-cause mortality (N=462)** | |  |  |
| **NPAR_exclude extreme** | 3.48 (2.17-5.58),  ***p<*0.0001** | 3.08 (1.87-5.05),  ***p<*0.0001** | 2.24 (1.35-3.74),  ***p=0.0019*** |
| **CVD-mortality (N=146)** | |  |  |
| **NPAR_exclude extreme** | 4.26 (2.09-8.68),  ***p<*0.0001** | 3.80 (1.74-8.30),  ***p=*0.0008** | 3.03 (1.33-6.90),  ***p*=0.0081** |

Model 1 was non-adjusted. Model 2 was adjusted for age, gender, BMI, race, education, tobacco use, and alcohol use. Model 3 was adjusted for age, gender, BMI, race, education, tobacco use, alcohol use, hypertension, hyperlipidemic, cardiovascular disease, history of diabetes, FPG, HbA1c, UA, TC, TC, HDLC, LDLC. Bold value indicates statistical significance.
